# Endogenous chondroitin extends the lifespan and healthspan in *C. elegans*

**DOI:** 10.1038/s41598-024-55417-7

**Published:** 2024-02-27

**Authors:** Yukimasa Shibata, Yuri Tanaka, Hiroyuki Sasakura, Yuki Morioka, Toshihiro Sassa, Shion Fujii, Kaito Mitsuzumi, Masashi Ikeno, Yukihiko Kubota, Kenji Kimura, Hidenao Toyoda, Kosei Takeuchi, Kiyoji Nishiwaki

**Affiliations:** 1 Department of Biomedical Sciences, Kwansei Gakuin University, 1 Gakuen Uegahara, Sanda, Hyogo 669-1330 Japan; 2 Department of Medical Cell Biology, School of Medicine, Aichi Medical University, Nagakute, Aichi Japan; 3 RIKEN Center for Developmental Biology, Kobe, Hyogo Japan; 4 Department of Bioinformatics, College of Life Sciences, Ritsumeikan University, Kusatsu, Shiga Japan; 5 Laboratory of Bio-Analytical Chemistry, College of Pharmaceutical Sciences, Ritsumeikan University, Kusatsu, Shiga Japan

**Keywords:** Aging, Chondroitin, ADAMTS protease, Basement membrane, Genetics, Molecular biology

## Abstract

Chondroitin, a class of glycosaminoglycan polysaccharides, is found as proteoglycans in the extracellular matrix, plays a crucial role in tissue morphogenesis during development and axonal regeneration. Ingestion of chondroitin prolongs the lifespan of *C. elegans*. However, the roles of endogenous chondroitin in regulating lifespan and healthspan mostly remain to be investigated. Here, we demonstrate that a gain-of-function mutation in MIG-22, the chondroitin polymerizing factor (ChPF), results in elevated chondroitin levels and a significant extension of both the lifespan and healthspan in *C. elegans*. Importantly, the remarkable longevity observed in *mig-22(gf)* mutants is dependent on SQV-5/chondroitin synthase (ChSy), highlighting the pivotal role of chondroitin in controlling both lifespan and healthspan. Additionally, the *mig-22(gf)* mutation effectively suppresses the reduced healthspan associated with the loss of MIG-17/ADAMTS metalloprotease, a crucial for factor in basement membrane (BM) remodeling. Our findings suggest that chondroitin functions in the control of healthspan downstream of MIG-17, while regulating lifespan through a pathway independent of MIG-17.

## Introduction

As animals age, tissue and organ malfunctions occur. For example, in old animals, including human being, declines in the ability of locomotion, sensation, the immune system, the digestive system, and regeneration are observed. Aging not only represents a significant risk factor for age-related diseases like cancer and atherosclerosis, but also exerts a substantial impact on an individual's overall quality of life. Aging was traditionally regarded as an irreversible process primarily driven by DNA mutations. However, the ability to clone mice successfully suggests that the accumulation of DNA mutations is not the primary cause of aging^1^. Aging is now considered a reversible process that can be treated as a disease. Activation of Sirtuins or AMPK, while inhibition of mTOR are known to suppress senescence intracellularly, and drugs targeting these factors are under investigation^2^.

The extracellular matrix (ECM) is a complex macromolecular structure enveloping tissues and organs. In general, the ECM is primarily composed of fibrous glycoproteins secreted by cells. There are many different types of ECM, including basement membrane (BM), cartilage, and invertebrate cuticle. Maintaining ECM homeostasis relies on a dynamic system regulated by a delicate equilibrium between synthesis, degradation, and reconstitution is crucial. Components of the ECM, such as collagen and chondroitin, decrease with age^3–5^. This age-related decline is associated with senescence-related collagen glycation and cross-linking, disrupting normal ECM reconstitution. Aging-related ECM degradation contributes to skin aging in mammals due to basement membrane (BM) damage, impacting the maintenance of hypodermal stem cells^6^. Additionally, the age-dependent decrease in collagen and chondroitin in cartilage is considered a factor in joint pain. Notably, in *C. elegans*, ECM collagen overexpression can inhibit senescence and extend lifespan^7^. Therefore, loss of ECM integrity is one of the causes of age-dependent disorders.

Chondroitin, which glycosylates proteoglycans in the ECM, is composed of N-acetylgalactosamine (GalNac) and glucuronic acid (GlcUA). The polymerization of chondroitin chains is catalyzed by the complex of chondroitin synthase (ChSy) and chondroitin-polymerizing factor (ChPF) complex^8^, which possesses both glucuronyltransferase and acetylgalactosamine-transferase activities. The length of the chondroitin chains varies depending on the specific combination of subunits of the chondroitin polymerizing enzyme complex^9^. Chondroitin proteoglycans not only fill the gaps in the ECM, but are also believed to play a role in signal transduction. Dysfunction of chondroitin synthase has been associated with abnormal development and impaired nerve regeneration^10,11^. Longevity associated with chondroitin ingestion has been observed in both humans and *C. elegans*^12,13^. Nevertheless, the roles of endogenous chondroitin in aging mostly remain to be elucidated.

The BM is a specialized sheet-like ECM that envelops tissues. The ADAMTS protease MIG-17 is secreted from the body wall muscle cells and localizes to the BMs of various tissues, and regulates cell migration and organ size in *C. elegans* depending through its protease activity^14–16^. MIG-17 is involved in the recruitment and modulation of BM molecules, including collagen IV and fibulin to regulate organogenesis^17–19^. While the function of MIG-17 in organogenesis has been investigated, its role in organismal aging remains to be elucidated.

In this study, we found that *mig-17* mutants exhibited accelerated senescence and that a gain-of-function mutation in MIG-22/ChPF, *mig-22(k185gf)*, suppressed this phenotype. We also demonstrated that *mig-22(k185gf)* mutants had an increased level of chondroitin and extended the lifespan and healthspan than wild-type animals. Genetic analyses suggest that MIG-17 regulates healthspan through chondroitin proteoglycans (CPGs), while lifespan is influenced by CPGs through mechanisms independent of MIG-17.

## Results

### Dominant mutation *k185* in MIG-22/ChPF

**Figure 1:**
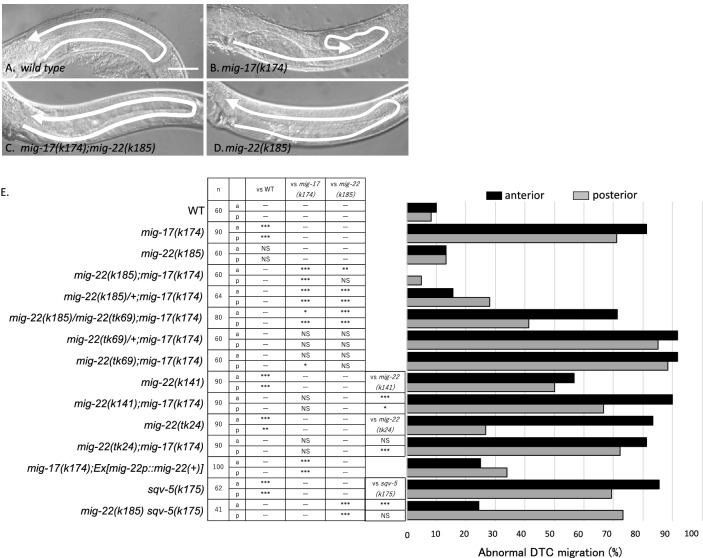
Suppression of the gonad migration defect of *mig-17* by *mig-22*(*k185gf*). (A-D) Nomarski images of young adult gonads in wild-type (**A**), *mig-17(k174)* (**B**), *mig-17(k174); mig-22(k185gf)* (**C**), and *mig-22(k185gf)* (**D**). Arrows indicate the shape of gonads. Dorsal is at the top. Bar: 50 μm. (**E**) Percentage of DTC migration defects. Black and gray bars represent defects in anterior and posterior gonad arms, respectively. *p*-values for Fisher’s exact test are indicated: ***p < 0.005, *p < 0.05, *NS* not significant, not determined.

During development of the *C. elegans* gonad, the gonadal leader cells, called distal tip cells (DTCs), migrate in a U-shaped pattern to form the U-shaped gonad arms. The *mig-17* mutants exhibit a misshapen gonad phenotype due to the meandering DTC migration^20^ (Fig. 1A,B). To elucidate the mechanism of DTC migration regulated by MIG-17, we screened and isolated a mutation *k185* as a genetic suppressor of the DTC migration defect in *mig-17(k174)* null mutants (Fig. 1A–C,E). The causative gene for *k185* was identified as *mig-22*, a gene previously known to be essential for DTC migration^10^. MIG-22 is a homologue of the human chondroitin polymerizing factor ChPF, which forms a complex with SQV-5/chondroitin synthase (ChSy)^8^. The *k185* mutation corresponds to a single nucleotide substitution from C to T, resulting in the L325P amino acid change. (Fig. S1). The amino acid Leucine corresponding to L325 of *C. elegans* MIG-22 is highly conserved among different species and is likely critical for protein function.

It has been reported that *mig-22* reduction-of-function (rf) alleles *k141* and *tk24*, as well as the deletion allele *tk69*, display meandering DTC migration defects similar to those observed in *mig-17* mutants^10^. In contrast, *mig-22(k185)* alone exhibited DTC migration phenotypes comparable to the wild type (Fig. 1D,E). While *mig-22(k185)* strongly suppressed the DTC migration defect of *mig-17(k174)* mutants, *mig-22(k185)/* + ;* mig-17(k174)* showed weak defects, suggesting that *k185* exhibits a semi-dominant effect (Fig. 1E). Since *k185* heterozygous with a wild-type allele showed a stronger suppressor activity compared to *k185* heterozygous with the deletion allele *tk69*, *k185* is a gain-of-function (gf) allele that enhances the function of the wild-type *mig-22* gene. *mig-22* reduction-of-function (rf) alleles *k141* and *tk24*, as well as the deletion allele *tk69*, failed to suppress the gonadal defects observed in *mig-17* mutants. We examined whether the overexpression of wild-type *mig-22* can suppress the *mig-17* defect. The extrachromosomal array carrying multicopy *mig-22* genes partially but significantly suppressed the gonadal defect in *mig-17* mutants (Fig. 1E). These results indicate that MIG-22 functions downstream of MIG-17.

### *mig-22(k185gf)* enhances chondroitin biosynthesis

**Figure 2:**
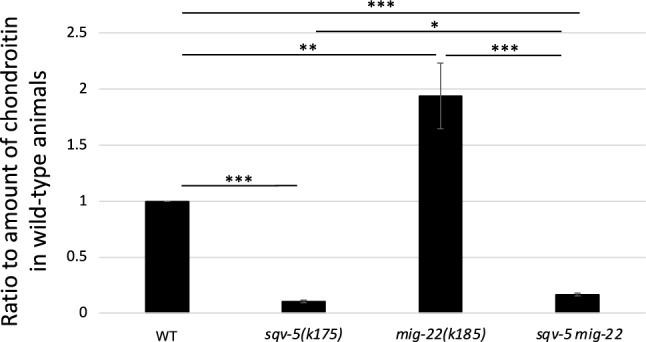
Amounts of chondroitin in mutants relative to wild type. Amounts of chondroitin were quantified by four independent sets of assays. The quantified values were averaged and shown as ratios relative to that of wild type. Error bars indicate standard error. *p*-values for *t* test are indicated: ***p < 0.005, **p < 0.01, *p < 0.05.

Since *mig-22* is required for chondroitin biosynthesis, we investigated whether *mig-22(k185gf)* increases the amount of chondroitin in vivo. We quantified chondroitin levels in *mig-22(k185gf*) mutants and the wild-type animals (Figs. 2, S2). Interestingly, the *mig-22(k185gf)* mutants exhibited chondroitin levels approximately twice as high as those in the wild-type animals, indicating that the *mig-22(k185gf)* mutation stimulates chondroitin biosynthesis.

The levels of chondroitin are known to decrease in the *sqv-5(k175)* mutant^10^, and we also observed a substantial reduction in chondroitin in the *sqv-5(k175)* mutant (Figs. 2, S2). The *k175* mutation represents a reduction-of-function mutation, and the enzyme activity may not be completely lost^10^. Therefore, we investigated whether the increase in chondroitin levels resulting from the *mig-22(k185gf)* mutation also occurs in the *sqv-5(k175)* mutant background. We found a slight but statistically significant increase in chondroitin levels in *sqv-5(k175); mig-22(k185gf)* when compared to *sqv-5(k175)* (Figs. 2, S2).

### *mig-22(k185gf)* extends lifespan and healthspan

**Figure 3:**
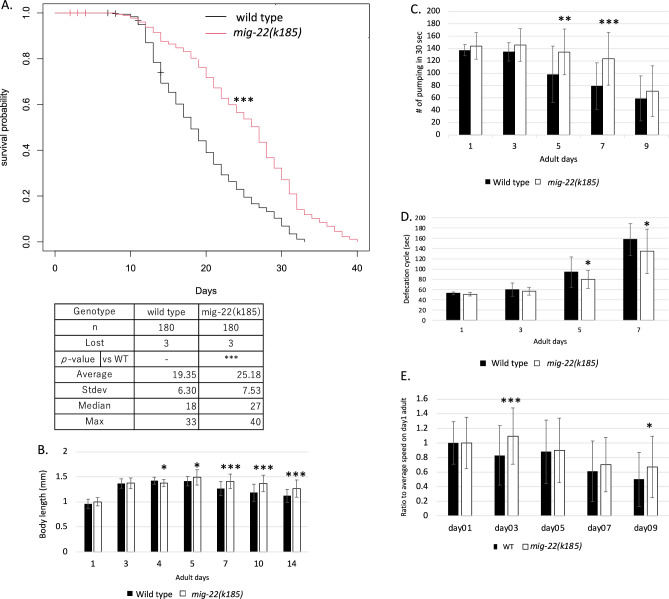
Suppression of aging by *mig-22(k185gf)* mutation. (**A**) The lifespan of wild-type and *mig-22(k185gf)*. The x axis shows days of adulthood. The y axis shows survival probability. The table displays the average, standard deviation, median, and maximum of lifespan. *p*-values for the logrank test are indicated: ***p < 0.005, – Not determined. (**B**–**E**) Body length (**B**), pumping rate (**C**), defecation cycIe (**D**), and mobility on *E.coli* (**E**) of wild-type and *mig-22(k185)* animals. Black and white bars indicate wild-type and *mig-22(k185)*, respectively. The x axis shows days in adulthood. The y axis shows body length (**B**), pumping rate in 30 s (**C**), defecation cycle (**D**), and ratio to average speed of day1 animals. *p*-values for *t* test against wild type are indicated: ***p < 0.005, **p < 0.01, *p < 0.05.

Since chondroitin sulfate intake has been shown to extend lifespan in *C. elegans*^13^, we investigated whether an increase in endogenous chondroitin affects lifespan. Interestingly, we observed that *mig-22(k185gf)* exhibited a longer lifespan compared to the wild type (*p* < 0.005 by logrank test) (Figs. 3A, S3). The lifespan of *mig-22(k185gf)* was 5.9 days (30.6%) longer on average than that of wild type, with a median increase of 9 days and a maximum lifespan extension of 7 days.

We also assessed whether *mig-22(k185gf)* had an impact on healthspan during adulthood in addition to lifespan. Specifically, we examined body length, pumping rate, and defecation cycle. In wild-type animals, the body length increased until day 4 and remained relatively stable through day 5. However, beyond day 7, a gradual decrease in body length was observed (Fig. 3B). Although the body length of *mig-22(k185gf)* animals increased as in the wild type, the shortening was significantly slower than the wild type. Notably, *mig-22(k185gf)* animals exhibited larger body sizes compared to the wild type at days 7, 10, and 14 (Figs. 3B, S4A,B).

The pharyngeal pumping rate is known to decrease with aging^21^. We examined changes in pumping rates along aging. In wild-type adults, the pumping rate was 137.3 ± 9.1 per 30 s on day 1, and this rate remained relatively consistent until day 3. Subsequently, at day 5, the pumping rate began to decline, reaching 59.0 ± 36.4 per 30 s by day 9 (Figs. 3C, S5A). However, in *mig-22(k185gf)* animals, no significant decrease in pumping rate was observed until day7. Consequently, *mig-22(k185gf)* maintained a faster pumping rate for a longer duration when compared to the wild type (Figs. 3C, S5A,B).

The defecation cycle elongates with aging^22^. In the wild type, the defecation cycle was 1.67 times longer in day 5 adults compared to day 1 adults (Fig. 3D). In *mig-22(k185gf)*, the defecation cycle also lengthened with age. However, *mig-22(k185gf)* exhibited a significantly shorter cycle compared to the wild type at day 5 and 7.

In *C. elegans*, the mobility of animals decreases with aging. In day 9 wild-type adults, mobility after tapping was almost half that of day 1 adults. The mobility of *mig-22(k185)* at day 1 adult was slower than that of wild-type animals (Fig S6). Therefore, we used the ratio to day 1 adult to compare the effect of aging on mobility (Fig. 3E). The downregulation of mobility with aging tended to be less in the *mig-22(k185)* background at day 9 adult than in the wild-type background. Taken together, these results indicate that the *mig-22(k185gf)* mutation, which increases endogenous chondroitin, slows aging by extending both lifespan and healthspan in the wild-type background.

### *mig-22(k185gf)* suppresses accelerated aging but not shortened lifespan of *sqv-5(k175)*

**Figure 4:**
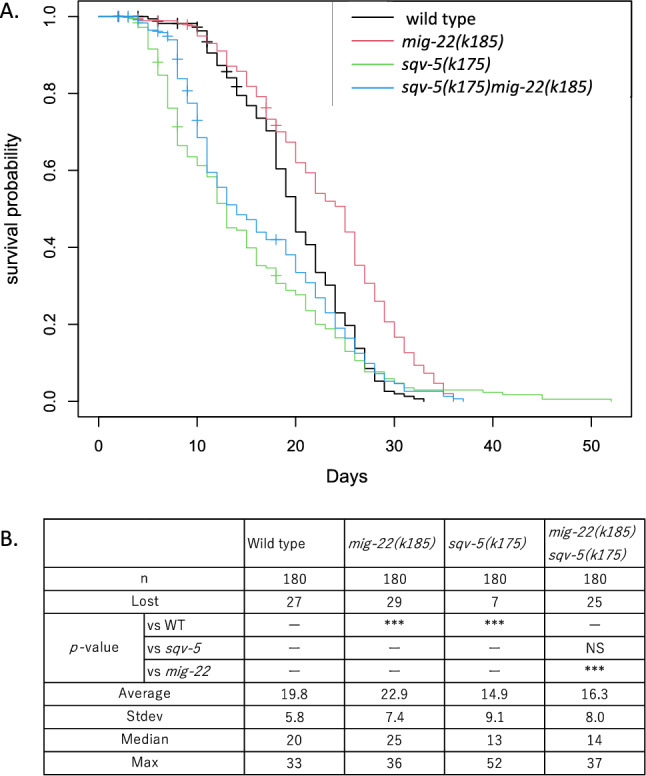
Lifespan extension by *mig-22(k185gf)* is dependent on *sqv-5*. (**A**) Comparison of lifespans of wild-type, *sqv-5 (k175)*, *mig-22(k185gf)*, and *sqv-5 (k175); mig-22(k185gf)* animals. The x axis shows lifespan in days of adulthood. The y axis shows the fraction of worms alive. (**B**) Table shows average, standard deviation, median, and maximum, of lifespan. *p*-values for log rank test are indicated: ***p < 0.005, **p < 0.01, *p < 0.05, *NS* not significant, not determined.

Mammalian ChPF and ChSy form a complex that synthesizes chondroitin polysaccharides^23^. The *C. elegans* homolog SQV-5/ChSy is required for gonad migration and embryogenesis^10,24^. However, the role of SQV-5 in aging is not clear. Therefore, we examined the lifespan of the *sqv-5(k175)* mutants that produce less chondroitin than wild type animals. We found that it was shorter than that of wild-type animals (*p* < 0.005 by logrank test) (Fig. 4A,B and S7). *sqv-5(k175)* had an average lifespan of 14.9 ± 9.1 days, which is 24.7% shorter than that of the wild type. When combined with *mig-22(k185gf)*, however, the shortened lifespan of *sqv-5(k175)* was not suppressed, indicating that the lifespan extension of *mig-22(k185gf)* depends on *sqv-5* (Fig. 4A,B).

**Figure 5:**
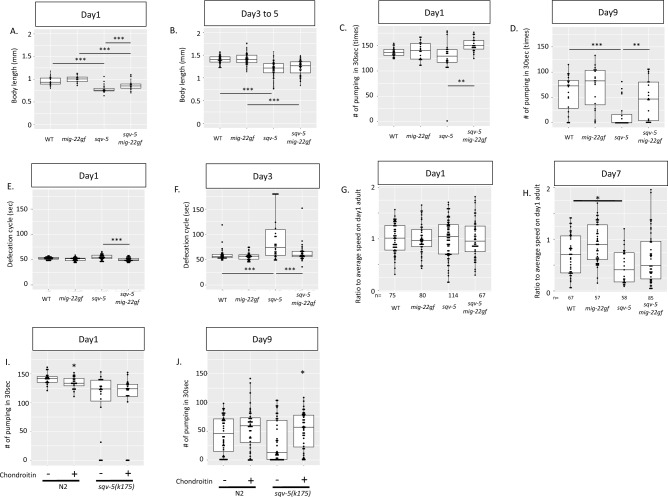
Suppression of accelerated aging in *sqv-5* mutants by *mig-22*(*k185gf*). Box and dot plots for body length (**A**,**B**), pumping rate (**C**,**D**), defecation cycle (**E**,**F**) and mobility on *E.coli* (**G**,**H**) of wild-type, *mig-22(k185)*, *sqv-5(k175)*, and *sqv-5(k175); mig-22(k185)* animals. Defecation cycle values exceeding 3 min were plotted as 180 s. (**I**,**J**) Box and dot plots for pumping rate of wild-type and *sqv-5(k175)* mutants are shown. Chondroitin sulfate supplementation is indicated at the bottom of the graph. *p*-values for *t*-test are indicated: ***p < 0.005, **p < 0.01, *p < 0.05.

The body length of the *sqv-5(k175)* mutants was shorter than that of wild-type animals at days 3 to 5 as well as at day 1, and this phenotype at day 1, but not at days 3 to 5, was suppressed by *mig-22(k185gf)* (Fig. 5A,B). The *sqv-5(k175)* mutants showed no abnormalities in the pumping rate at day 1, but exhibited a slower pumping rate compared to the wild type at day 9 (Figs. 5C, S5A,C). The pumping defect was suppressed by *mig-22(k185gf).* At day 9, although almost half of *sqv-5(k175)* mutants stopped pumping, *sqv-5(k175); mig-22(k185gf)* double mutants persisted pumping behavior (Figs. 5D, S5C,D). Similarly, *sqv-5(k175)* showed no defecation abnormality at day 1, but significantly longer defecation cycle at day 3, which could be suppressed by *mig-22(k185gf).* About 20% of the *sqv-5(k175)* mutants had a defecation cycle longer than 3 min, but no such animals were observed in *sqv-5(k175); mig-22(k185gf)* (Fig. 5E,F). *sqv-5(k175)* mutants showed larger downregulation of the mobility with aging than wild-type animals (Fig. 5G,H). In contrast, in *sqv-5(k175); mig-22(k185)* double mutants, degree of downregulation is similar to that of wild-type animals. These results indicate that *mig-22(k185gf)* can partially suppress the defects in healthspan, but not in the lifespan of *sqv-5(k175)*.

If slower pumping in *sqv-5(k175)* mutants is caused by the reduction of chondroitin, supplementation of chondroitin can rescue the slower pumping in *sqv-5(k175)* background. On day1 of adulthood, supplementation of chondroitin had no effect in the *sqv-5(k175)* background, although it caused a slight reduction in the pumping rate in the wild-type background (Fig. 5I). On day 5 of adulthood, a significant rescue of slower pumping was observed by the supplementation of chondroitin in the *sqv-5(k175)* background (Fig. 5J).

We also measured brood size and found no correlation between aging and brood size (Fig S9). For example, *mig-22(k185gf)* mutation suppressed small brood size of *sqv-5(k175)*, but not affect the short lifespan of *sqv-5(k175)*.

### *mig-22(k185gf)* suppresses shorter body length and slower periodic behavior in aged *mig-17* mutants

**Figure 6:**
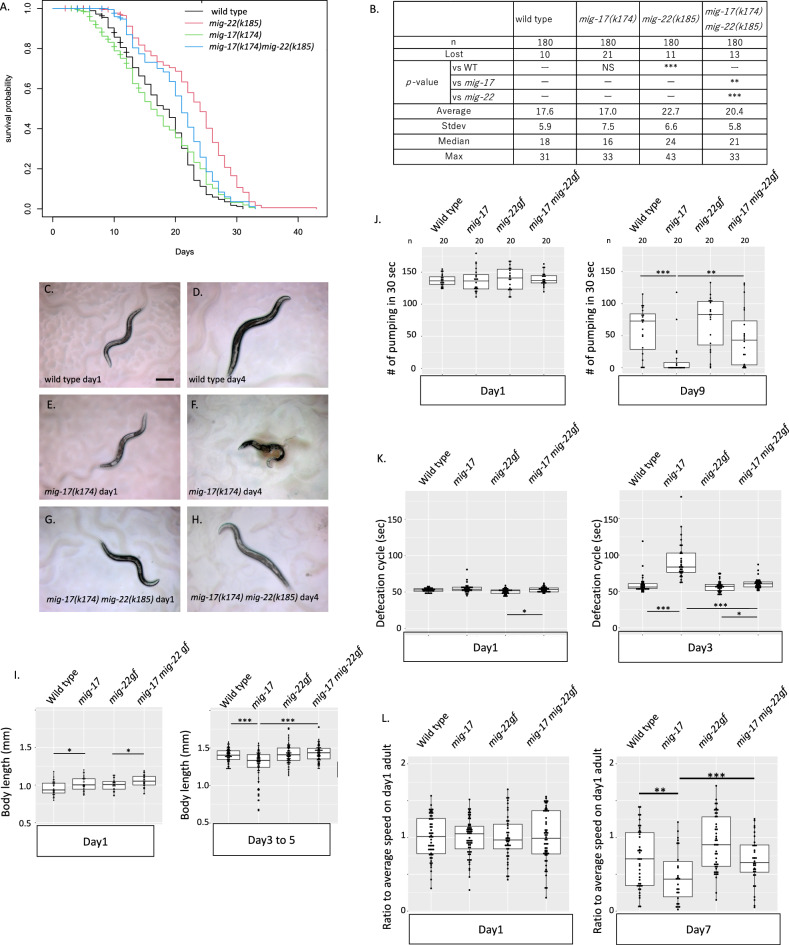
Suppression of shorter body length and slower periodic behavior in aged *mig-17* mutants by *mig-22(k185gf)* mutation. (**A**) The lifespan of wild-type, *mig-17(k174)*, *mig-22(k185gf)*, and *mig-17(k174); mig-22(k185gf)*. The x axis shows days of adulthood. The y axis shows survival probability. (**B**) Table shows average, standard deviation, median, and maximum of lifespan. *p*-values for logrank test are indicated: ***p < 0.005, **p < 0.01, *NS* not significant, not determined. Images of wild-type (**C**,**D**), *mig-17(k174)* (**E**,**F**), *mig-17(k174); mig-22(k185)* (**G**,**H**) of day 1 (**C**,**E**,**G**) and day 4 (**D**,**F**,**H**) adult. (**I**–**L**) A box and dot plot that shows body length (**I**), pumping rate (**J**), defecation cycle (**K**) and mobility on *E.coli* (**L**) of wild-type, *mig-17(k174)*, *mig-22(k185)*, and *mig-17(k174); mig-22(k185)* animals. *p*-values for *t* test are indicated: ***p < 0.005, **p < 0.01, *p < 0.05.

Since *mig-22(k185gf)* suppressed the *mig-17(k174)* DTC migration phenotype, we explored the possibility that *mig-17* is also implicated in aging. We examined lifespan, body length, pumping, and defecation cycle in a *mig-17(k174)* background. The lifespan of *mig-17(k174)* mutants was comparable to that of wild type (Figs. 6A,B, S10). On the other hand, *mig-22(k185); mig-17(k174)* double mutants had a longer lifespan than *mig-17(k174)* or wild type, but a shorter lifespan than *mig-22(k185gf)*. These results indicate that *mig-17* is not responsible for the wild-type lifespan. However, *mig-17* partially suppresses *mig-22* long-lived phenotype.

Although the body length of *mig-17* mutants was slightly longer than that of the wild type at day 1, it became significantly shorter than wild type at days 3 to 5 (Figs. 6C–F,I, S4A,E). *mig-22(k185gf)* suppressed the shortening phenotype of *mig-17* (Figs. 6E–I, S4B,E,F). Although a fraction of *mig-17* animals showed very short body sizes of less than 1 mm at day 3 to 5, *mig-22(k185gf)* also suppressed this phenotype (Figs. 6I, S4F).

The pumping rate, defecation cycle, and mobility of *mig-17(k174)* mutants were similar to those of the wild-type animals in day 1 adults, suggesting that *mig-17* mutants can form fully functional pharynx, intestine, and locomotive system (Figs. 6J–L, S5A,E, S6C). However, *mig-17* mutants showed significantly slower pumping at day 9 and altered defecation behaviors at day 3 compared to wild-type animals. Downregulation of mobility at the day 7 adult was larger in *mig-17(k174)* mutants than that of wild type animals. These phenotypes of *mig-17* were suppressed by *mig-22(k185gf)* (Figs. 6J–L, S5A,B,E,F, S6C,D). These results indicate that *mig-22(k185gf)* suppresses multiple phenotypes observed in aged *mig-17* mutants, including alterations in the body length and periodic behaviors.

## Discussion

In this study, we have isolated a gain-of-function mutation of the *mig-22* gene, which encodes a chondroitin polymerizing factor. The *mig-22(k185gf)* mutation suppressed the gonadal defects of the *mig-17* mutant. Furthermore, we observed that the *mig-22(k185gf)* mutation extended lifespan and healthspan when compared to the wild type. Conversely, the reduction-of-function mutation *k175* in the *sqv-5* gene, encoding ChSy, led to premature aging and a shortened lifespan. Both MIG-22 and SQV-5 are homologs of mammalian ChPF and ChSy, which form a complex to synthesize chondroitin chains^23^.

The chondroitin levels in *mig-22(k185gf)* were increased twofold compared to those of the wild type, whereas the levels in *sqv-5(k175rf)* were markedly reduced compared to the wild type. We also previously reported a significant reduction in chondroitin levels in *mig-22(k141rf)*^10^. In this study, we observed a slight but significant increase in chondroitin levels in the presence of *mig-22(k185gf),* even in the *sqv-5(k175rf)* background, suggesting that the residual ChSy activity contributes to this increase.

Mammalian ChSy binds to ChPF, and the chondroitin polymerization activity of ChSy is higher when ChSy and ChPF are co-expressed^23^. While ChPF is homologous to ChSy in its primary structure, but ChPF itself has a weak chondroitin chain polymerizing activity. In mammals, multiple ChPF and ChSy complexes result in varying chondroitin chain lengths depending on the specific combination of these complexes^9^. Notably, the knockout of ChPF and ChPF2 in cancer cells has been reported to shorten the length of chondroitin chains^25^. Therefore, it is plausible that the altered chondroitin chain length, rather than the quantity of chondroitin chains attaching to the core proteins, contributes to the increased levels in *mig-22(k185gf)* and the decreased levels in *mig-22(rf)* and *sqv-5(rf)* mutants.

*mig-22(k185gf)* was identified as a strong suppressor of the DTC migration defect associated with the *mig-17(k174)* null allele. This suggests that the elevated chondroitin levels may serve to compensate for the loss of MIG-17-dependent proteolysis within the BM. Given that MIG-17 plays a role in ensuring proper attachment of the DTCs to the body wall^14^, it is conceivable that the increased chondroitin levels in proteoglycans residing within the body wall BM or in the gonadal BM could potentially restore the appropriate adhesion between these tissues in the absence of MIG-17.

**Figure 7:**
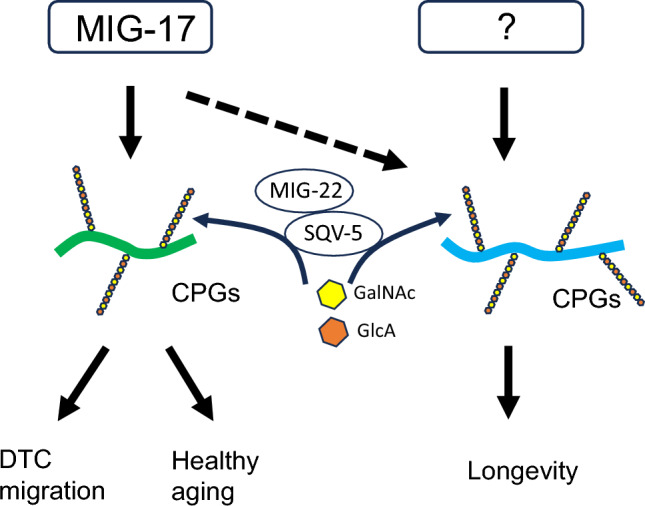
Model for the CPG-mediated regulation of aging. MIG-17-dependent proteolysis activates CPG signaling within the basement membrane (BM). The CPGs then activate the downstream pathway to regulate gonadal DTC migration and healthy aging. CPGs in the BM or other ECM mediate unknown signals to promote longevity. MIG-17 is required for longevity when the amount of chondroitin modification of CPGs exceeds wild-type levels (dotted arrow).

Since *mig-17* mutants did not affect the lifespan in an otherwise wild-type background, it is reasonable to conclude that *mig-17* does not play a role in the regulation of lifespan. However, phenotypes observed in aged adults, including shortening of body length and the decline in the pumping rate and defecation cycle, were observed earlier in *mig-17* mutants than in wild-type animals. These observations suggest that MIG-17 likely governs the BM physiology in multiple tissues to support normal aging. Based on our genetic analyses, we propose that CPGs act downstream of MIG-17, promoting DTC migration and contributing to healthy aging in a manner dependent on the amount of chondroitin attached (Fig. 7). Given that ADAMTS proteases in mammals often degrade proteoglycans, it is plausible that the substrates of MIG-17 might include CPGs^26^.

*mig-22(k185gf)* exhibited a longer lifespan than the wild type, whereas the lifespan of *sqv-5(k175rf)* was significantly shorter compared to the wild type. Notably, *mig-22(k185gf)* failed to suppress the shortened lifespan and shorter body length of *sqv-5(k175rf).* However, *mig-22(k185gf)* could suppress the accelerated aging phenotypes of periodic behaviors in the *sqv-5(k175rf)* background. These results suggest that chondroitin levels play a crucial role in the regulation of both lifespan and healthspan. It is possible that different thresholds of chondroitin levels may exist to control distinct outputs for lifespan and healthspan. It is also noteworthy that an increase in chondroitin levels beyond wild-type levels extends both lifespan and healthspan. MIG-17 appears to be required for lifespan extension only when excessive amount of chondroitin is produced. In summary, these findings indicate that the regulation of healthspan is under the MIG-17 pathway, while a separate regulatory pathway independent of MIG-17 is implicated in the regulation of lifespan (Fig. 7).

CPGs are known to be involved in various critical processes, including ECM assembly, cell adhesion, cell migration, proliferation, and nerve regeneration^10,11,24,27^. There is existing evidence that chondroitin levels decrease with aging^28^. Moreover, chondroitin has been reported to prevent the induction of aging-related phenomena. For instance, in the nervous system of mice, reduced chondroitin synthesis is associated with accelerated age-related memory decline^29^. In human studies, a cohort ranging from 50 to 76 years old showed that chondroitin intake was linked to reduced mortality, implying a potential lifespan-extending effect^12^. Furthermore, in *C. elegans*, feeding chondroitin sulfate has been shown to extend lifespan^13^. This indicates a consistent correlation between chondroitin intake and extended lifespan extension is observed across different species. Nevertheless, the precise role of endogenous chondroitin in the regulation of aging is remains enigmatic.

In this study, we demonstrated a correlation between chondroitin levels in vivo and lifespan and healthspan. Chondroitin modifies over 20 core proteins in *C. elegans*^30^. Different chondroitin proteoglycans may be involved in various aging-related processes. The *mig-22(k185gf)* mutation represents a senescence-suppressing genetic polymorphism. The Leucine residue at position 325, corresponding to the *mig-22(k185gf)* mutation site is conserved across different species. It would be intriguing to investigate whether genetic polymorphisms in ChPF proteins, similar to *mig-22(k185gf)*, have lifespan-extending effects in mammals, including humans. Further research is essential to elucidate the precise mechanisms underlying aging in relation to chondroitin levels of CPGs.

## Methods

### Strains and genetic analysis

Culture, handling and ethyl methanesulfonate (EMS) mutagenesis of *C*. *elegans* were conducted as described^31^. Worms were cultured at 20 °C. The following mutations and markers were used in this work: *mig-17(k174)*^14^*, mig-22(k141), mig-22(tk69), mig-22(tk24),* and *sqv-5(k175)*^10^. The suppressor mutation *k185* was genetically mapped using single-nucleotide polymorphism mapping to linkage group III, with a *mig-17(k174)* mutant strain in the CB4856 background^32^. Whole genome sequence analysis comparing *mig-17(k174)* and *mig-17(k174); k185* in the mapped region identified a single mutation in the coding sequence corresponding to *mig-22*.

### Microscopy

Gonad migration phenotypes were scored using a Nomarski microscope (Axioplan 2; Zeiss). Analysis of gonadal phenotypes was performed in the young-adult stage, as previously described^14^.

Measure body length, photographs of animals on an agar plate were taken using a 5 × objective lens. The measurements were conducted using ImageJ software. Each strain was sampled with 30 individuals per day.

### Analysis of chondroitin levels

Wild-type, *mig-22(k185), sqv-5(k175),* and *sqv-5(k175); mig-22(k185)* animals were grown on NGM plates at 20 °C and collected as mixed-stage animals using M9 buffer. The animals were washed twice with M9 buffer and cultured for 1 h at room temperature. After removing the supernatant, 10 ml of acetone was added. The analysis of the CS chain analysis was conducted by enzymatic treatment and HPLC-based quantification as described previously^11,27^.

### Behavioral analyses

Defecation cycle and pumping rate were analyzed as previously described^33^. To synchronize the animals for the analysis of defecation cycle, pumping rate or body size, we collected young adults with no fertilized eggs. To distinguish them from their progeny, they were transferred them to a new plate every two days while they were laying eggs. The pumping rate was measured for 30 s, and the average of three consecutive measurements was calculated. If the defecation cycle exceeded 3 min, further measurements were discontinued. The sample size was 20 for pumping and 30 for the defecation cycle.

Mobility assays were conducted by measuring the speed of animals after tapping plates with *E*. *coli*. Movies were recorded for 90 s after tapping, and images were analyzed using the MTrack2 plugin in ImageJ.

Supplementation of chondroitin was performed as described^13^. Animals were maintained for two generations before measuring pumping rate.

### Analysis of lifespan

Animals were synchronized for lifespan analysis by collecting L4 stage individuals. Lifespan was analyzed as previously described^34^.

### Analysis of brood size

Brood size is measured as described^35^.

### Supplementary Information

Supplementary Figure S1.

Supplementary Figure S2.

Supplementary Figure S3.

Supplementary Figure S4.

Supplementary Figure S5.

Supplementary Figure S6.

Supplementary Figure S7.

Supplementary Figure S8.

Supplementary Figure S9.

Supplementary Figure S10.

Supplementary Legends.

## Data Availability

Strains are available upon request. The authors state that all the data necessary for confirming the conclusions presented in the article are represented fully within the article.
