# Supplementary figures and images for: Endogenous chondroitin extends the lifespan and healthspan in C. elegans
Source: Sci Rep. 2024 Feb 27;14:4813. doi: 10.1038/s41598-024-55417-7 (PMC10899230; doi:10.1038/s41598-024-55417-7)

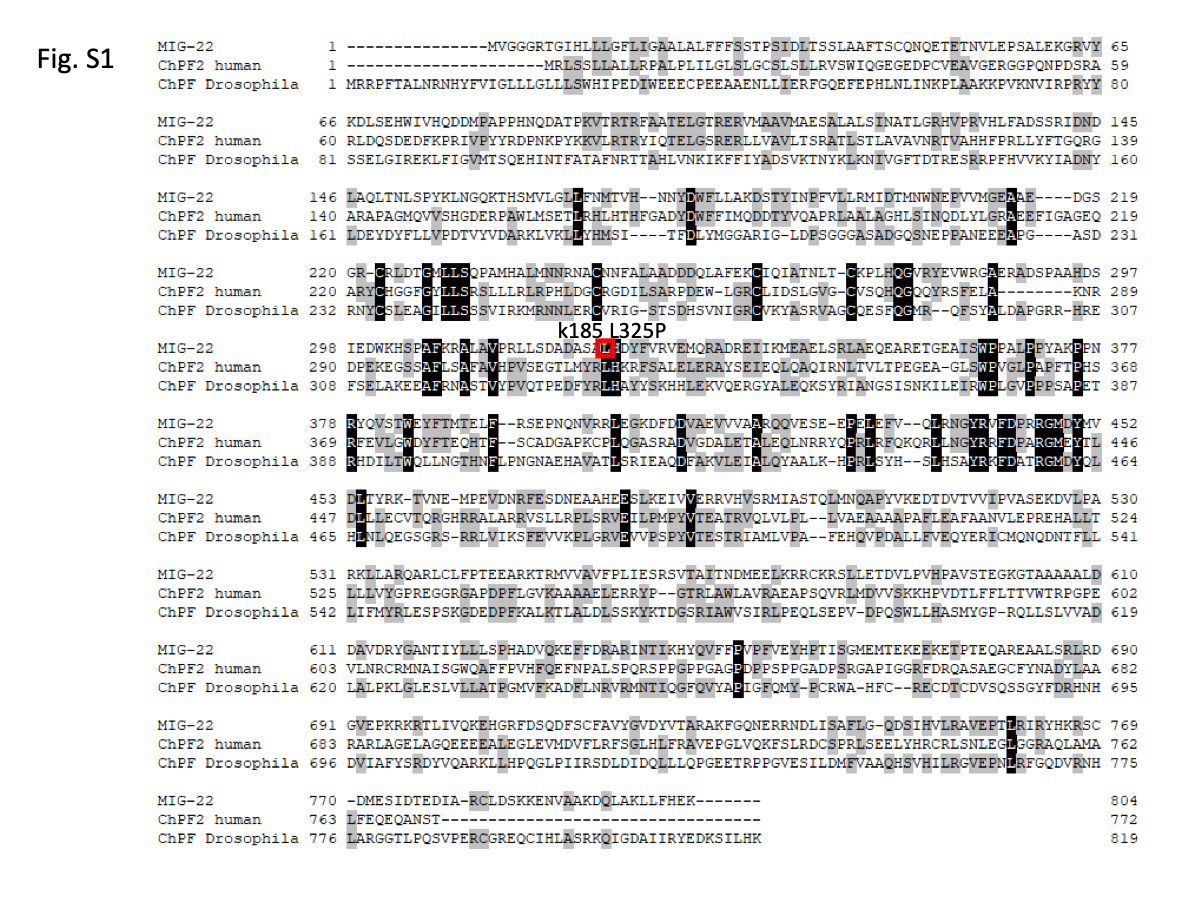

Supplement: Supplementary file 1 — Supplementary Figure S1. [file 41598_2024_55417_MOESM1_ESM.tiff]

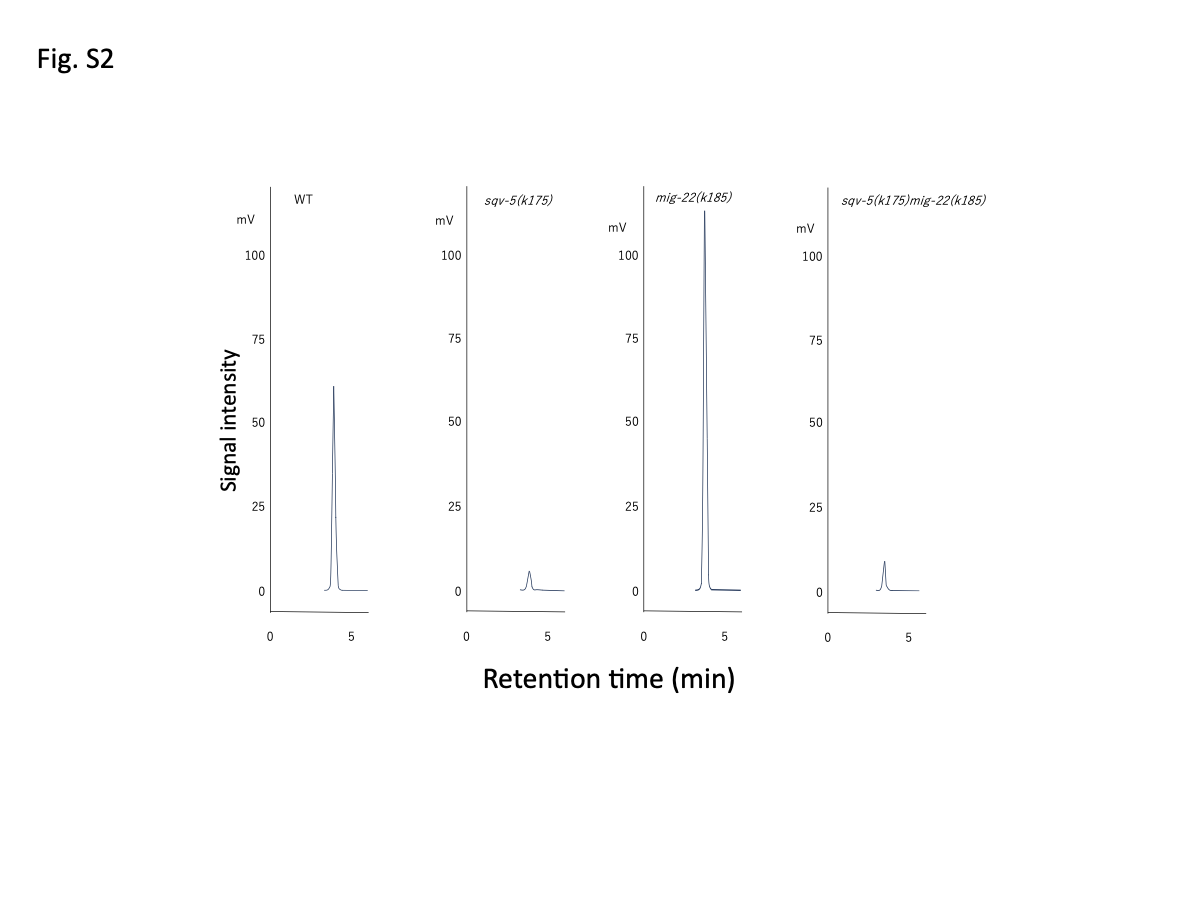

Supplement: Supplementary file 2 — Supplementary Figure S2. [file 41598_2024_55417_MOESM2_ESM.tiff]

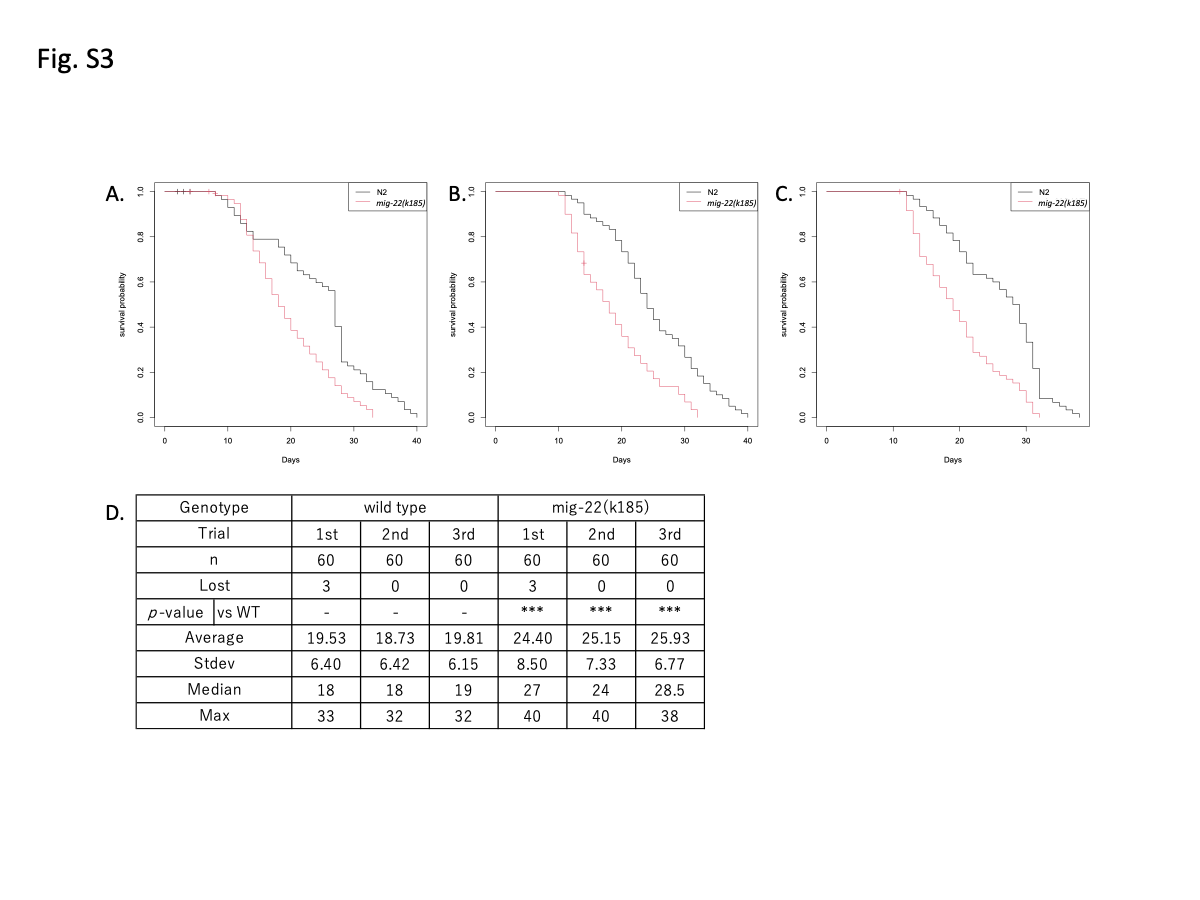

Supplement: Supplementary file 3 — Supplementary Figure S3. [file 41598_2024_55417_MOESM3_ESM.tiff]

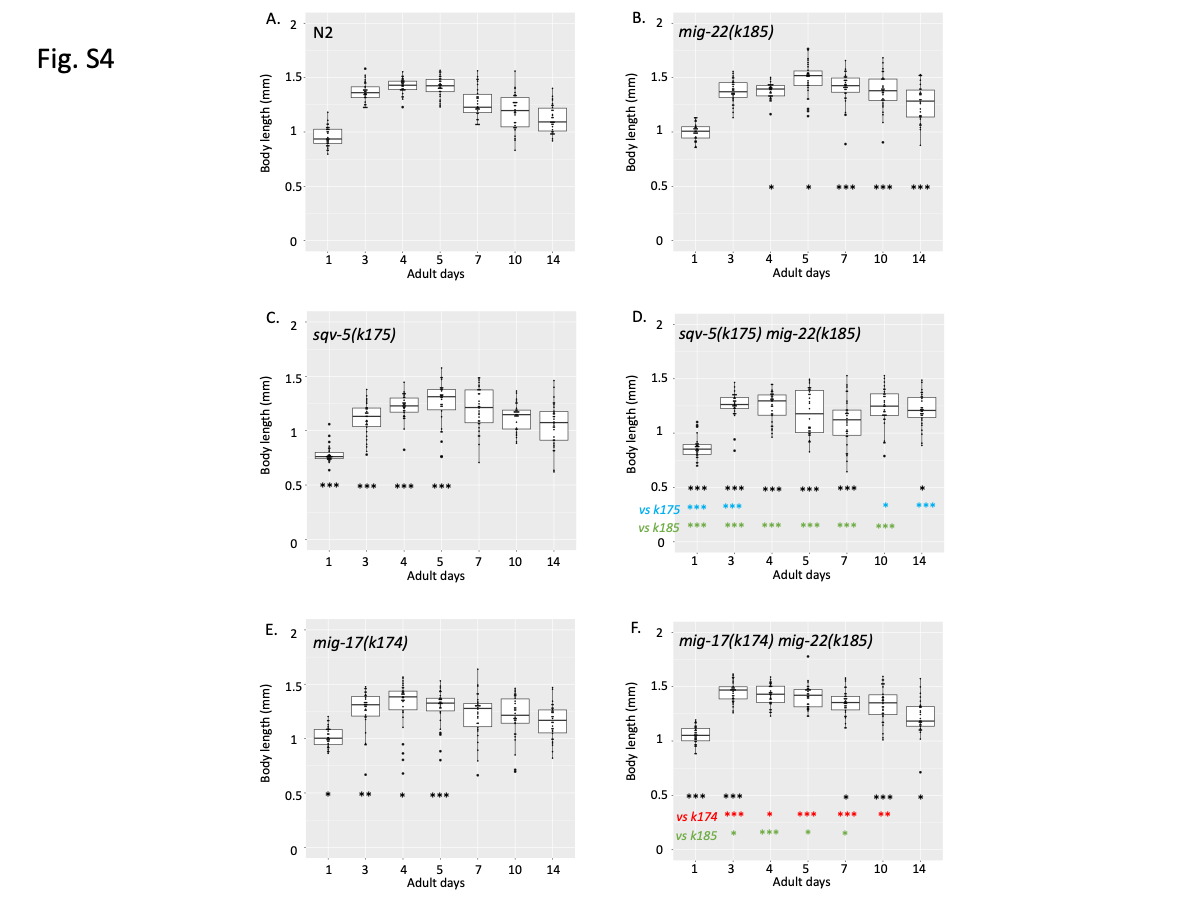

Supplement: Supplementary file 4 — Supplementary Figure S4. [file 41598_2024_55417_MOESM4_ESM.tiff]

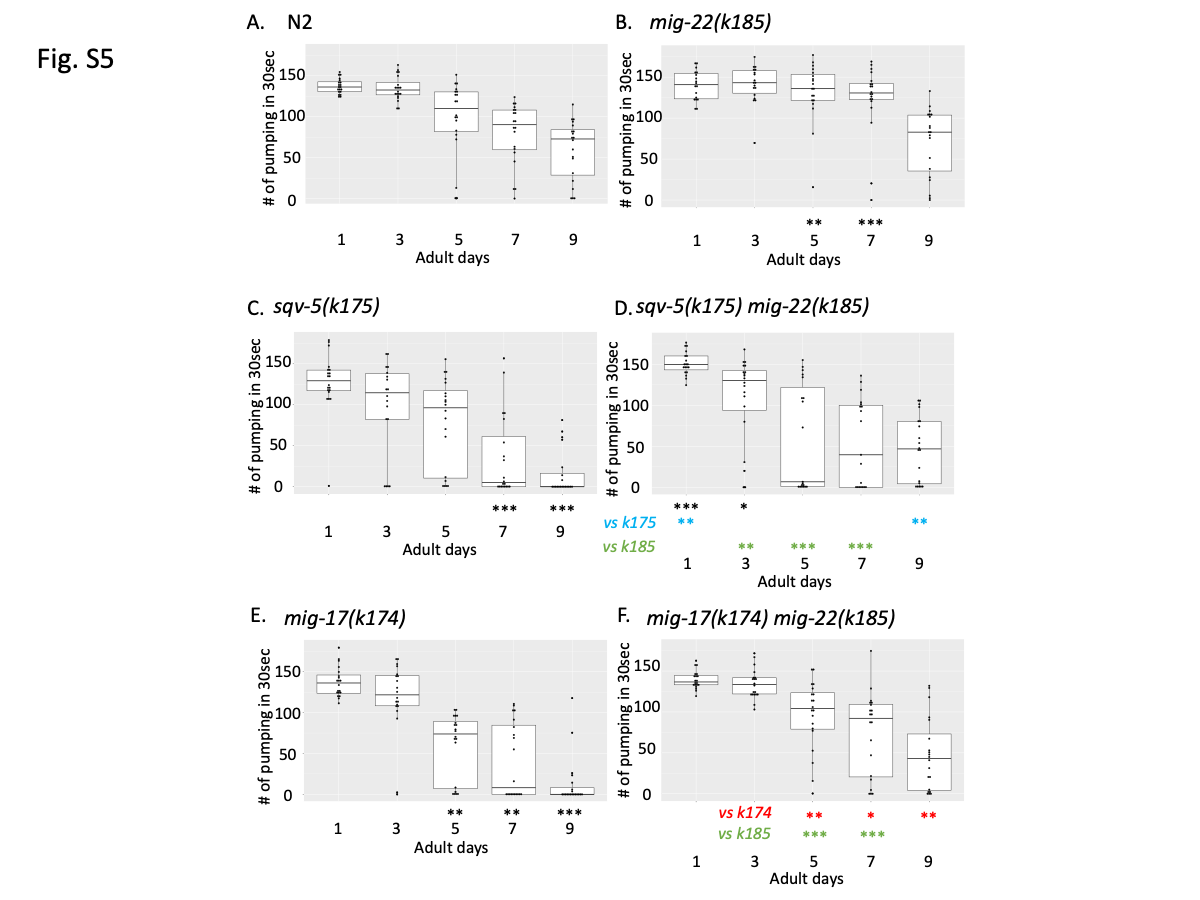

Supplement: Supplementary file 5 — Supplementary Figure S5. [file 41598_2024_55417_MOESM5_ESM.tiff]

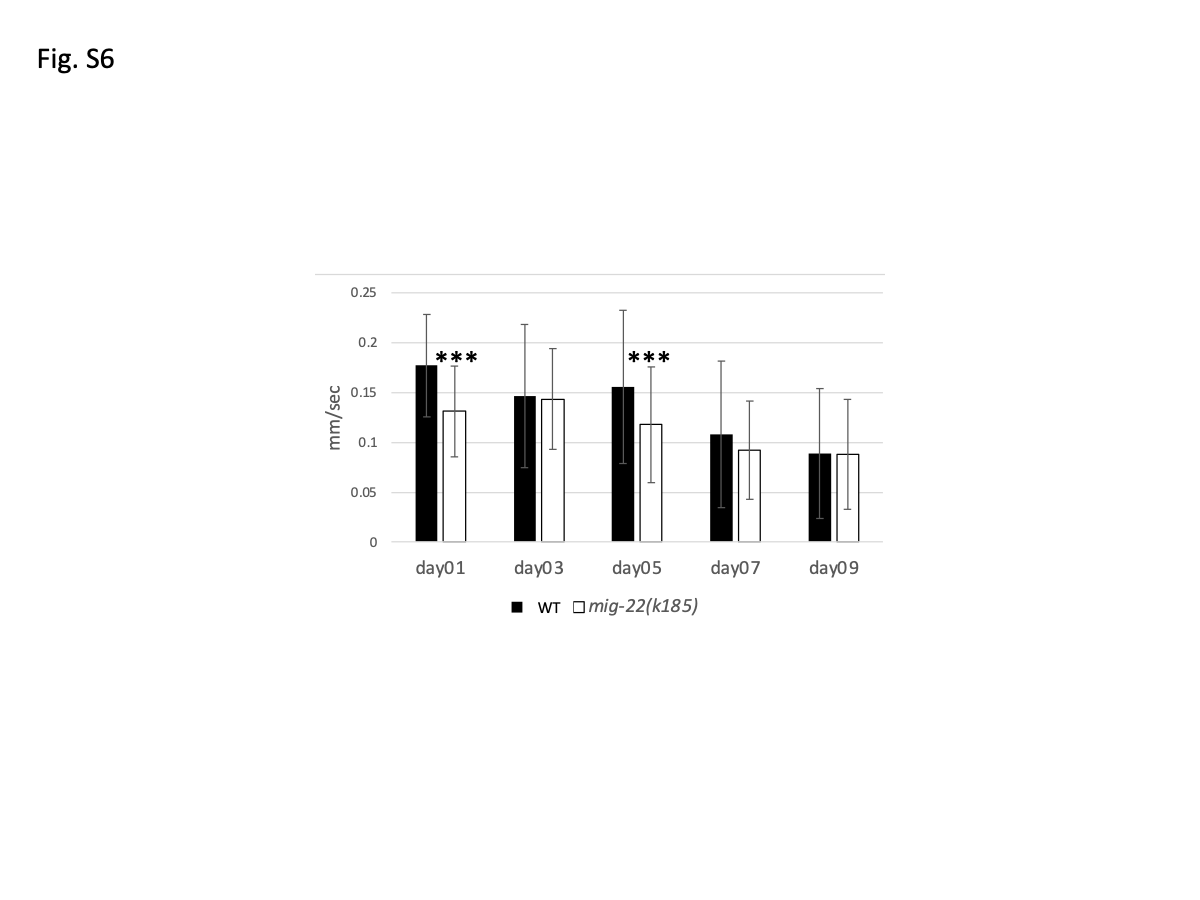

Supplement: Supplementary file 6 — Supplementary Figure S6. [file 41598_2024_55417_MOESM6_ESM.tiff]

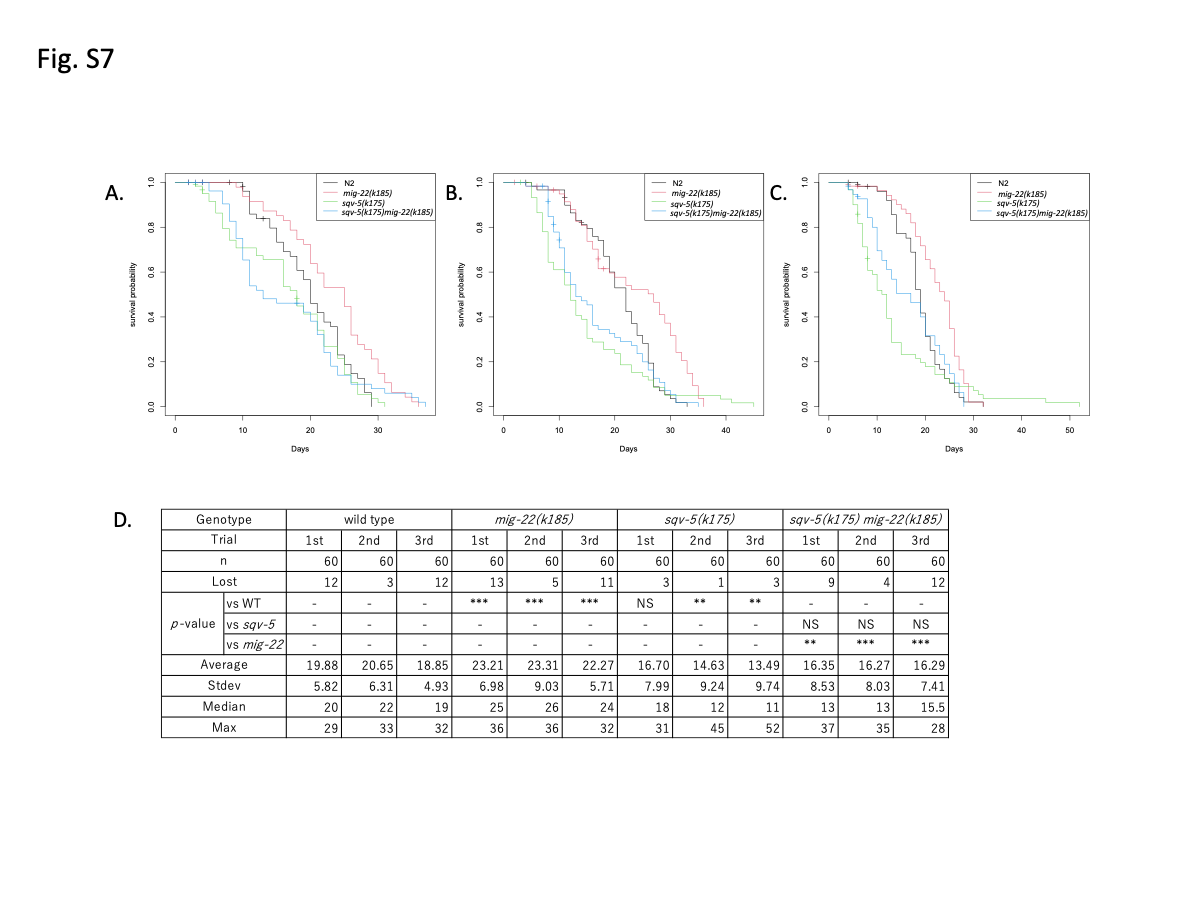

Supplement: Supplementary file 7 — Supplementary Figure S7. [file 41598_2024_55417_MOESM7_ESM.tiff]

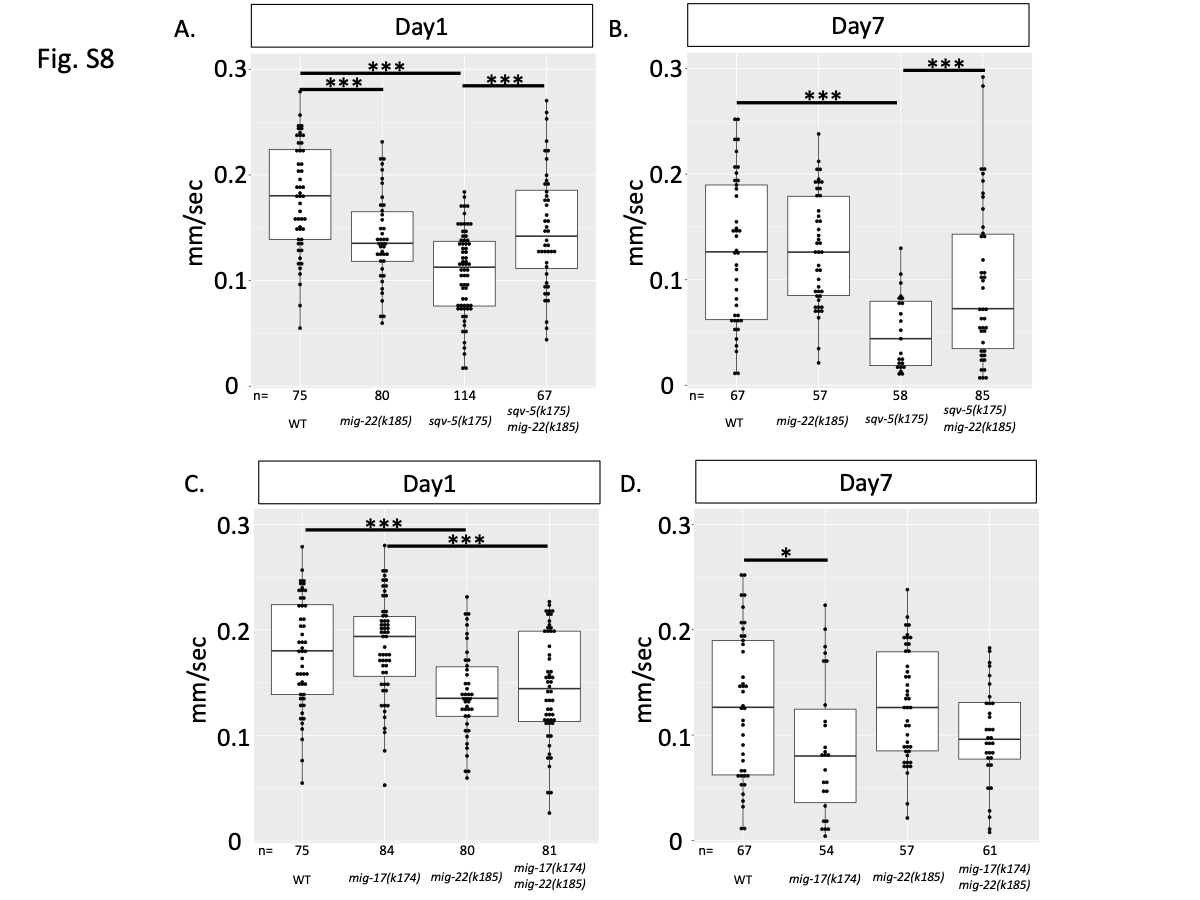

Supplement: Supplementary file 8 — Supplementary Figure S8. [file 41598_2024_55417_MOESM8_ESM.tiff]

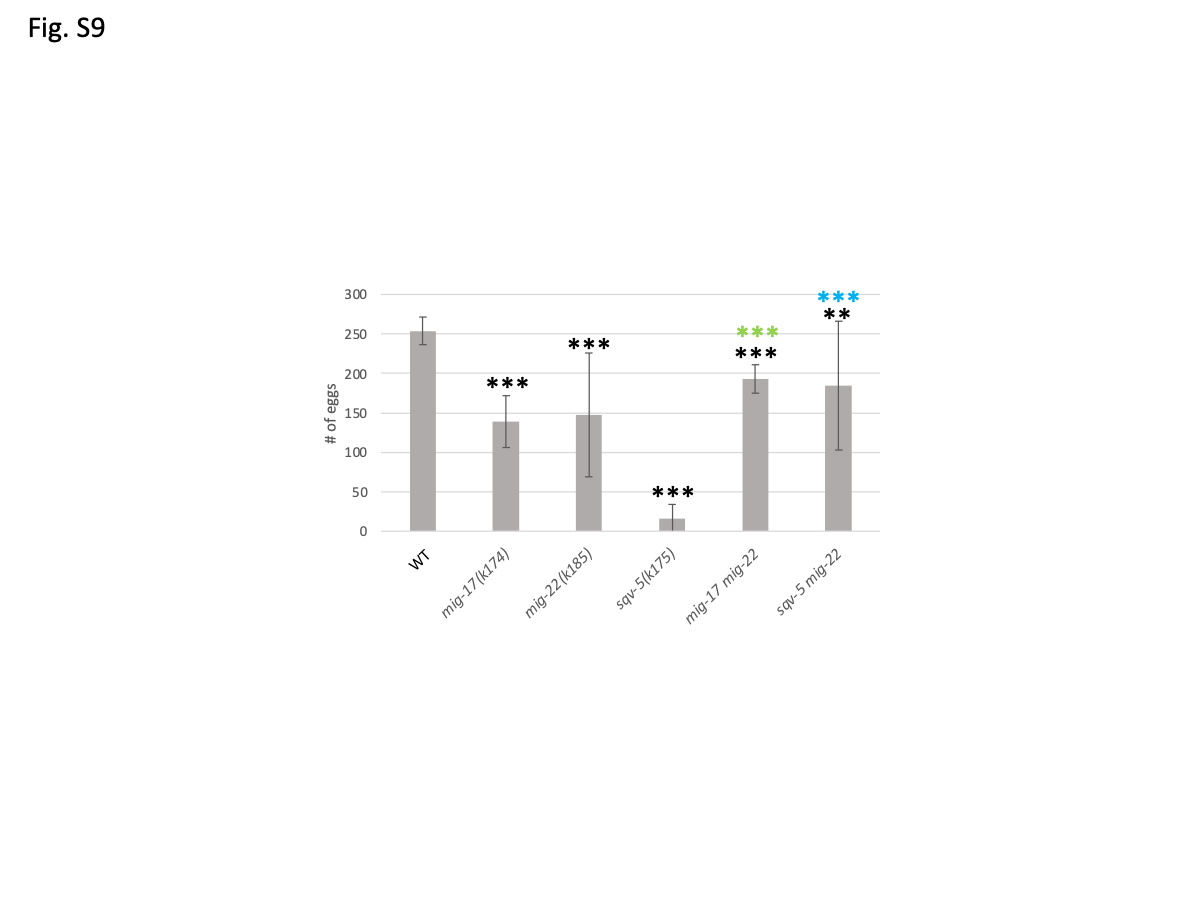

Supplement: Supplementary file 9 — Supplementary Figure S9. [file 41598_2024_55417_MOESM9_ESM.tiff]

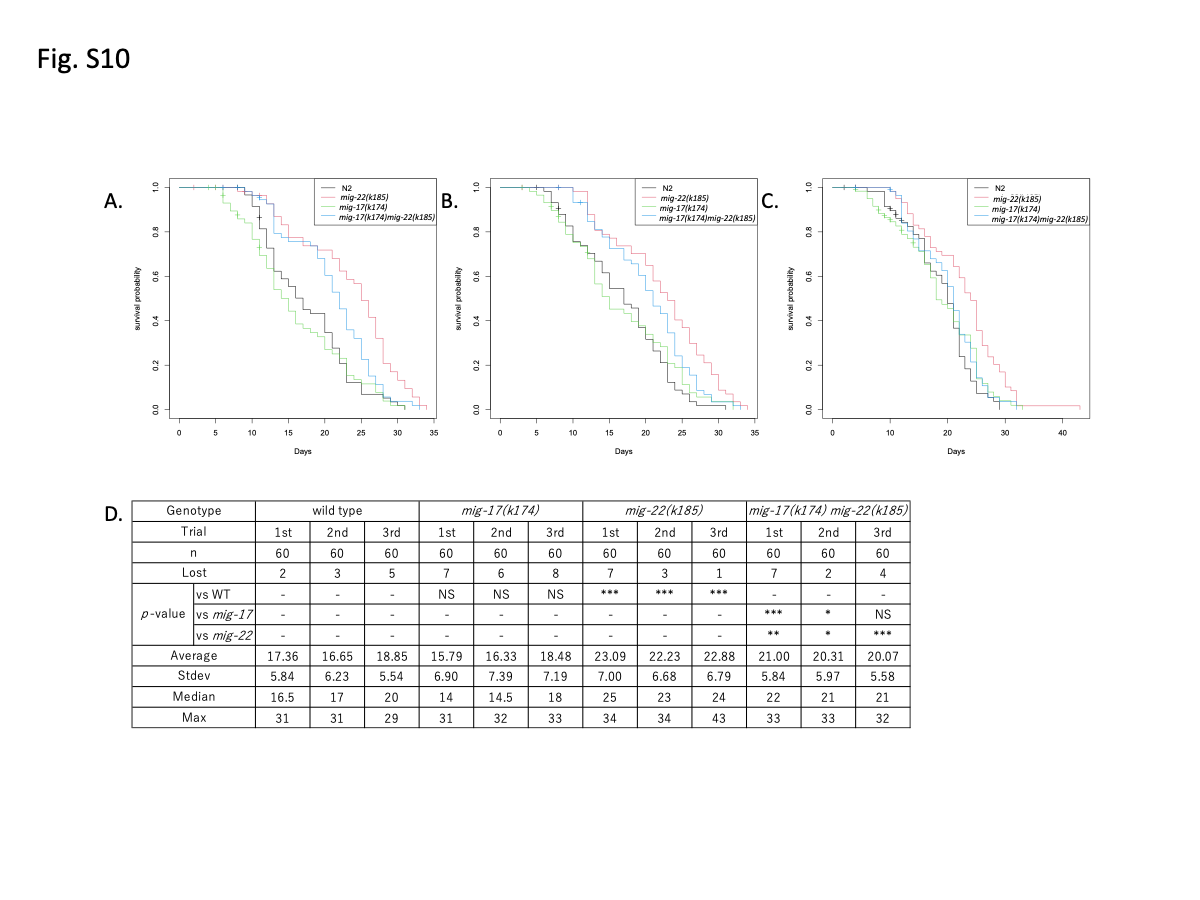

Supplement: Supplementary file 10 — Supplementary Figure S10. [file 41598_2024_55417_MOESM10_ESM.tiff]
