# Supplementary material for: Endogenous chondroitin extends the lifespan and healthspan in C. elegans
Source: Sci Rep. 2024 Feb 27;14:4813. doi: 10.1038/s41598-024-55417-7 (PMC10899230; doi:10.1038/s41598-024-55417-7)
Supplement: Supplementary file 11 — Supplementary Legends. [file 41598_2024_55417_MOESM11_ESM.docx]

**Legend of supplemental figures**

**Figure S1 Alignment of ChPF proteins**

Identical residues and similar residues are shaded in black and gray, respectively. The position of the k185 L325P mutation is indicated by the red box.

**Figure S2 Quantification of chondroitin levels**

HPLC profiles of disaccharides from chondroitin in one of the four sets of experiments using wild-type, *sqv-5(k175)*, *mig-22(k185gf)* and *sqv-5(k175); mig-22(k185gf)* samples.

**Figure S3** **Lifespan extension by *mig-22(k185gf)***

(A-C) Comparison of lifespans between wild-type and *mig-22(k185gf)* animals. The x axis represents lifespan in days of adulthood. The y axis shows the fraction of worms alive. The 1^st^ (A), 2^nd^ (B) and 3^rd^ (C) trials are indicated separately. (D) Table shows average, standard deviation, median, and maximum, of lifespan. *p*-values for logrank test are indicated: ****p* < 0.005, ***p* < 0.01, **p* < 0.05, NS Not significant, – Not determined.

**Figure S4** **Suppression of body length defects by *mig-22(k185gf)***

Box and dot plots indicate the body length of wild-type (A), *mig-22(K185)* (B), *sqv-5(k175)* (C), *sqv-5(k175); mig-22(k185)* (D), *mig-17(k174)* (E), and *mig-17(k174) mig-22(k185)* (F) animals. *p*-values for t-test against WT, *mig-17(k174), mig-22(k185)*, and *sqv-5(k175)* are indicated in the graph by black, red, green, and blue asterisks, respectively: ****p* < 0.005, ***p* < 0.01, **p* < 0.05.

**Figure S5** **Suppression of pumping rate defects by *mig-22(k185gf)***

Box and dot plots indicate the number of pumping in 30 seconds in wild-type (A), *mig-22(K185)* (B), *sqv-5(k175)* (C), *sqv-5(k175); mig-22(k185)* (D), *mig-17(k174)* (E), and *mig-17(k174) mig-22(k185)* (F) animals. *p*-values for t-test against WT, *mig-17(k174), mig-22(k185)*, and *sqv-5(k175)* are indicated in the graph by black, red, green, and blue asterisks, respectively: ****p* < 0.005, ***p* < 0.01, **p* < 0.05.

**Figure S6 Suppression of slow movement in aged adult by *mig-22(k185gf)***

Bar plot indicates the moving speed of wild-type and *mig-22(k185)* animals. *p*-values for t-test against WT are indicated in the graph: ****p* < 0.005.

**Figure S7** **The effect of *mig-22(k185gf)* and *sqv-5(k175)* on lifespan**

(A-C) Comparison of lifespans of wild-type, *sqv-5 (k175)*, *mig-22(k185gf)*, and *sqv-5 (k175); mig-22(k185gf)* animals. The x axis shows lifespan in days of adulthood. The y axis shows the fraction of worms alive. The 1^st^ (A), 2^nd^ (B) and 3^rd^ (C) trials are indicated separately. (D) Table shows the average, standard deviation, median, and maximum, of lifespan. *p*-values for logrank test are indicated: ****p* < 0.005, ***p* < 0.01, **p* < 0.05, NS Not significant, – Not determined.

**Figure S8 The effects of *mig-22(k185gf), sqv-5(k175)*, and *mig-17(k174)* on mobility**

Box and dot plots indicating the mobility of wild-type, *mig-22(K185)*, *sqv-5(k175)*, *sqv-5(k175); mig-22(k185)*, *mig-17(k174)*, and *mig-17(k174) mig-22(k185)* animals. *P*-values for t-test are indicated: ****p* < 0.005, ***p* < 0.01, **p* < 0.05.

**Figure S9 The effect of *mig-22(k185gf)*, *sqv-5(k175)* and *mig-17(k174)* on brood size**

A bar graph indicating brood size. *p*-values for t-test against WT, *mig-22(k185)*, and *sqv-5(k175)* are indicated in the graph by black, green, and blue asterisks, respectively: ****p* < 0.005, ***p* < 0.01. n=12.

**Figure S10** **The effect of *mig-22(k185gf)* and *mig-17(k174)* on lifespan**

(A-C) Comparison of lifespans of wild-type, *mig-17(k174)*, *mig-22(k185gf)*, and *mig-17 (k174); mig-22(k185gf)* animals. The x axis shows lifespan in days of adulthood. The y axis shows the fraction of worms alive. 1^st^ (A), 2^nd^ (B) and 3^rd^ (C) trial is indicated separately. (D) Table shows average, standard deviation, median, and maximum, of lifespan. *p*-values for logrank test are indicated: ****p* < 0.005, ***p* < 0.01, **p* < 0.05, NS Not significant, – Not determined.
